# Development of a Japanese version of the patient perceptions of deprescribing – Short form

**DOI:** 10.1002/jgf2.733

**Published:** 2024-10-06

**Authors:** Mio Kushibuchi, Kenya Ie, Masaki Takahashi, Amy M. Linsky, Steven M. Albert

**Affiliations:** ^1^ Faculty of Public Health & Policy London School of Hygiene & Tropical Medicine London UK; ^2^ Department of General Internal Medicine St. Marianna University School of Medicine Kawasaki‐shi Kanagawa Japan; ^3^ Department of General Internal Medicine, Department of Internal Medicine Kawasaki Municipal Tama Hospital Kawasaki‐shi Kanagawa Japan; ^4^ Division of Medical Informatics St. Marianna University School of Medicine Kawasaki‐shi Kanagawa Japan; ^5^ General Internal Medicine Center for Healthcare Organization and Implementation Research (CHOIR), and New England Geriatric Research Education and Clinical Center (GRECC); VA Boston Healthcare System Boston Massachusetts USA; ^6^ General Internal Medicine Boston University Chobanian & Avedisian School of Medicine Boston Massachusetts USA; ^7^ Department of Behavioral and Community Health Sciences University of Pittsburgh Graduate School of Public Health Pittsburgh Pennsylvania USA

**Keywords:** deprescribing, geriatrics, patient perspectives, polypharmacy, primary health care, questionnaire

## Abstract

**Background:**

Deprescribing is a critical component of clinical practice, especially in geriatric medicine. Nevertheless, the attributes of patients who are prepared for, interested in, and could potentially benefit from deprescribing have not been well examined. The Patient Perceptions of Deprescribing (PPoD) evaluates patients' overall readiness for deprescribing and is complemented by an 11‐item validated short form (SF‐PPoD). The objective of this study was to develop a Japanese version of the SF‐PPoD and assess its reliability and validity within Japanese older adults with polypharmacy.

**Methods:**

The SF‐PPoD was translated, back‐translated, and assessed in a cognitive interview. We conducted a cross‐sectional survey with 196 patients aged 65 years or older with five or more medications using the Japanese version to test for reliability and validity. We examined internal consistency and construct validity to determine if the Japanese sample responses reproduce the two subscales in the original SF‐PPoD. Finally, we examined intra‐person replicability using the intraclass correlation coefficient, in which 100 participants were invited and 93 participated.

**Results:**

118 males and 78 females, with a mean age of 79.2 [SD 6.5] years, completed the survey. Confirmatory factor analysis of the Japanese version of SF‐PPoD revealed satisfactory structural validity with two‐dimensional structure, “Motivation for Deprescribing” and “Primary Care Physician Relationship.” Cronbach's alpha showed good internal consistency, and test–retest demonstrated acceptable intra‐rater reliability.

**Conclusions:**

We developed and validated a Japanese version of SF‐PPoD with an 11‐item, two‐dimensional structure consistent with the original SF‐PPoD. This scale may facilitate shared decision‐making for medication optimization among older adults living with multimorbidity.

## Graphical abstract

The study successfully developed and validated a Japanese version of the Short‐Form Patient Perceptions of Deprescribing (SF‐PPoD) with 11 items and a two‐dimensional structure. The Japanese SF‐PPoD demonstrated satisfactory reliability and validity, supporting its use in facilitating shared decision‐making for medication optimization among older adults with polypharmacy.

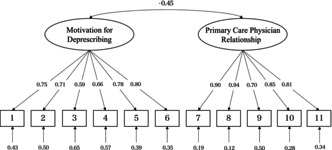

## 1 BACKGROUND

Deprescribing, defined as “the process of tapering or stopping drugs, aimed at minimizing polypharmacy and improving outcomes”,^1^ is a critical component of clinical practice, especially in the context of geriatric care. Some harms reported to be associated with polypharmacy – commonly defined as five or more chronic medications – are adverse drug events, non‐adherence,^2^ reduced cognitive and physical capability,^3^ and multiple geriatric syndromes such as falls,^4^ dementia,^5^ and depression.^6^ Polypharmacy is also associated with increased mortality and shorter overall survival among older patients.^4^

Various interventions have been attempted to reduce polypharmacy, but the results for clinical outcomes are mixed.^7^, ^8^, ^9^ A recent narrative review highlights the importance of patient education, among other interventions, such as using criteria for deprescribing,^10^ medication review, and clinician education.^7^ The importance of patient readiness for deprescribing has been underscored repeatedly, and previous studies report that many patients want fewer medications.^11^, ^12^, ^13^ Still, patient‐clinician relationship factors, such as trust, reliance on expertise, shared decision‐making, and balancing multiple providers, may be barriers to patients' deprescribing readiness.^11^ Another systematic review described barriers to patients' decisions to reduce medications, such as medication appropriateness, deprescribing process, potential influences of ceasing a medication, and fear of deprescribing.^14^ Nevertheless, the attributes of patients willing to consider deprescribing and who could potentially benefit from it have not been well studied, partly because of the lack of a standardized measure to ascertain such characteristics in various settings.

The Patient Perceptions of Deprescribing (PPoD), a 30‐item questionnaire, evaluates patients' overall readiness for deprescribing^15^ and is complemented by an 11‐item validated short form (SF‐PPoD).^16^ The original version, developed by Linsky et al. using respondents receiving care from the US Veterans Health Administration (VA), was comprised of variables derived from several questionnaires, including Beliefs about Medications,^17^ trust in the physician,^18^ CollaboRATE,^19^ Patients attitudes toward deprescribing (PATD),^13^ and autonomy preference index.^20^

The original version consisted of three domains – conflicting views of medication, the importance of the patient‐provider relationship, and experience with medication discontinuation.^15^ For the short‐form version, items on the original scale were regrouped into two domains – motivation for deprescribing and primary care provider relationship.^16^ The strength of the SF‐PPoD is that it is a short and psychometrically valid measure of patients' readiness to deprescribe, which can be used in clinical and research settings. Further, it is explicitly intended for use among older adults with polypharmacy, and it addresses the important concepts of motivation and patient‐provider relationship.^12^, ^13^, ^17^, ^18^, ^19^, ^20^

Japan is one of the world's most aged societies. In one study, half of patients in Japan aged 85 years or older had polypharmacy,^21^ and the number of medications increased with age, with a mean of 5.74 per patient among adults aged 80–89 years.^22^ The Japanese healthcare system ensures free access to any healthcare facility without the referral of a primary care physician. While the prevalence of polypharmacy is higher among patients who visit multiple hospitals,^23^ Japanese patients receiving care in a community‐oriented primary care clinic, rather than hospitals, were less likely to meet criteria for polypharmacy.^24^ This evidence suggests that primary care may be vital in decreasing polypharmacy in Japan. Thus, evaluating patients' readiness to deprescribe should be meaningful to Japanese primary care physicians and their patients.

To the best of our knowledge, there is currently no validated scale for assessing deprescribing readiness among patients within the Japanese healthcare context. Some studies have used PATD to evaluate patients' attitudes toward deprescribing in Japan,^25^ but the measure was not validated for use in this context. Regarding patient self‐management, the 13‐item version of the Patient Activation Measure is translated and validated among Japanese young adult cancer survivors,^26^ but the measurement does not explicitly ask about deprescribing. Therefore, the objective of this study was to develop a Japanese version of the SF‐PPoD and assess its reliability and validity in Japanese older adults with polypharmacy.

## 2 METHODS

### 2.1 Study design

This study was comprised of two components: the initial translation and cognitive interview pilot testing of the SF‐PPoD, followed by a subsequent cross‐sectional study using the newly developed Japanese version of the SF‐PPoD to test its reliability and validity. Written informed consent was obtained from all participants. The study protocol was approved by the Institutional Ethical Committee of St. Marianna University School of Medicine (No. 5593).

### 2.2 Setting and participants

For the pilot testing of the provisional Japanese version, a panel of 10 Japanese community‐dwelling adults 65 years or older with five or more concurrent prescription medications participated in the cognitive interview. The panel was recruited from a group of primary care patients who volunteer to contribute to care quality improvement by providing patients' perspectives.

**FIGURE 1:**
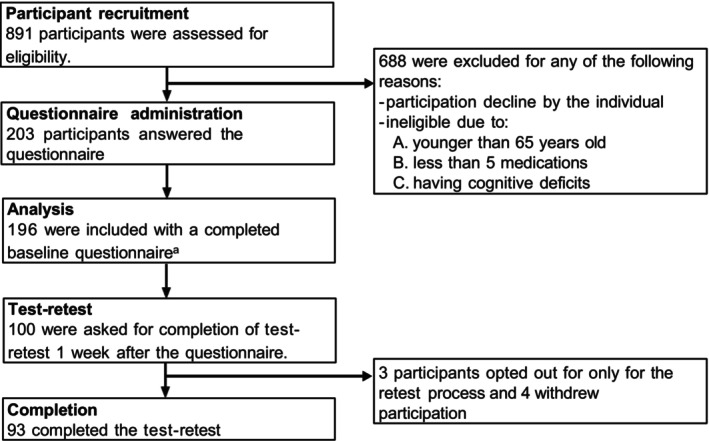
Flowchart of the cross‐sectional study. ^a^200 were initially included, but 4 completely withdrew participation at a later stage of the study, and thus 196 were included in the full dataset.

For the cross‐sectional study, we recruited participants using convenience sampling from a primary care clinic and a community hospital in Kawasaki City, Japan, from May to October 2023. The patient inclusion and exclusion flowchart for this part is shown in Figure 1. Community‐dwelling adults who visited the institutions were eligible to participate if they (1) were 65 years or older, (2) visited the institution within the previous 6 months, (3) communicated in Japanese and provided written consent to participate, (4) received five or more regular prescription medications, and (5) indicated they received a majority of their care from a primary care physician (PCP). Patients were excluded if they had a diagnosis of dementia, were admitted to a hospital within 30 days prior to study participation, or if the study lead deemed it inappropriate to participate. Regular medication was defined as any orally administered prescription medication with a duration of 28 days or longer documented in either the participant's electronic health record or in the medication record (“okusuri‐techo”: documents printed by the pharmacy and maintained by patients) at the time of enrollment.

### 2.3 Development of the Japanese version of SF‐PPoD

The short form Patient Perceptions of Deprescribing was translated into a provisional Japanese version based on recommended approaches from the International Society for Pharmacoeconomics and Outcomes Research (ISPOR) guideline^27^ and leading US federal agencies.^28^, ^29^, ^30^ Our approach allowed informed cultural adaptation rather than simple translation, as recommended for health survey instruments.^29^

#### 2.3.1 Initial translation and back‐translation

The SF‐PPoD was initially translated into Japanese by a PCP familiar with polypharmacy and deprescribing, who is a native Japanese speaker and English speaker (KI). Following recommendations, the translation aimed to maintain conceptual equivalence to the original version rather than literal word‐for‐word translation. The initial Japanese version was then translated back into English by a bilingual Japanese and English speaker (MK).

#### 2.3.2 Translation team review

The back‐translated English version was jointly reviewed by a team of Japanese and English speakers to review conceptual and cultural equivalence. The review included the research team (KI, MK, AML, SMA), supplemented by additional bilingual Japanese‐English speakers. The team discussed any discrepancies, and further revisions were made until the team reached consensus.

#### 2.3.3 Pre‐testing and cognitive interview pilot

The provisional Japanese version of the SF‐PPoD was administered to the panel of 10 patients for feedback regarding interpretability and acceptability. Participants' think‐aloud commentary was elicited as they completed questions to help establish how questions were perceived and possible misunderstandings in question items or instructions that may require revision. We assessed the items using the Manchester Cultural Adaptation Reporting Questionnaire, which specifies benchmarks for cultural adaptation.^31^

### 2.4 Cross‐sectional survey

The participants were asked to respond to the Japanese version of the SF‐PPoD. In addition, the following variables were collected to describe participant characteristics: the use of long‐term care insurance, education, place of residence, subjective relative health, and comorbidities. The use of long‐term care insurance was categorized as not receiving benefits, support levels 1–2, care levels 1–2, and care levels 3–5. Support levels indicate that one is largely independent but needs partial assistance; care levels indicate that support is required continuously, where people with care levels 1–2 are largely housebound and those with care level 5 are bedbound.^32^, ^33^, ^34^ The highest education obtained was categorized into junior high school, high school, or higher education (including vocational school, universities, and college). The place of residence was recorded as either a long‐term care facility or a home. Subjective relative health was obtained by asking: “Compared to peers of your own age, how do you consider your health?” Options were a 5‐point Likert scale ranging from very good health to bad health. Participants reported diagnoses of cancer, hypertension, diabetes mellitus, dyslipidemia, myocardial infarction and angina, chronic heart failure, asthma, chronic obstructive pulmonary disease (COPD), chronic renal failure, liver disease or cirrhosis, dementia, and depression. The participants were also asked whether they had any emergency department visits, hospitalizations, or falls in the previous 6 months.

### 2.5 Validation process utilizing cross‐sectional survey response

Using the cross‐sectional study response, we assessed the reliability and validity of the Japanese version of the SF‐PPoD. All validation processes complied with the COnsensus‐based Standards for the selection of health Measurement Instruments (COSMIN) Study Design checklist for patient‐reported outcome measurement instruments.^35^ Confirmatory factor analysis (CFA) was used to determine if the Japanese sample responses reproduce the two subscales identified in the US sample.^16^ Model fitness was evaluated using the comparative fit index (CFI), Tucker‐Lewis index (TLI), root mean square error of approximation (RMSEA), and standardized root mean square residual (SRMR). As a representation of models with a good fit, thresholds of CFI > 0.95, TLI > 0.95, RMSEA < 0.06, and SRMR < 0.08 were employed.^36^ Cronbach's alpha coefficient was used to assess internal consistency. Subsequently, we examined test–retest reliability with 93 participants who agreed to respond to the questionnaire twice with a minimum of 1 week between responses. Reliability was assessed using the intraclass correlation coefficient. To test for convergent validity, the participants were asked whether they had talked about changing their medications with their PCP in the previous 6 months. Among those who answered “yes,” we also asked whether a change was made according to the consultation, and among those who had a change, we asked whether this change in medication resulted in a change in their health status. These answers were categorized into three consultation‐based groups: “did not consult about medication change,” “consulted but had no medication change (or increase in the number of medications),” and “consulted and had a reduction of medication.” We evaluated whether the mean scores for the three consultation‐based groups were different using ANOVA. We also compared the means for those who did not consult versus those who consulted (regardless of medication change) using the Wilcoxon signed rank test. We hypothesized that although there is no gold standard to test for readiness for deprescribing, this question theoretically measures a related construct.

All statistical analyses were conducted using R version 4.3.2.

## 3 RESULTS

### 3.1 Translation and back translation

The original SF‐PPoD, the translated Japanese version, and the back‐translated English version are shown in Table S1. Subscales 1 and 2 measure ‘motivation for deprescribing’ and ‘primary care provider relationship’ and consist of 6 and 5 items, respectively. In the cognitive debriefing with 10 sample participants, we discussed that in the Japanese context, primary care providers are always physicians, and thus the Japanese SF‐PPoD would be better comprehended if it used the term primary care physician. Content validity, especially face validity, was ascertained by the research team, which included researchers in this area, PCP practicing in Japan, and the developer of the original PPoD‐SF. After thorough revision, a finalized version was approved. The finalized version of the Japanese PPoD‐SF is shown in Table S2.

### 3.2 Validation process

A total of 203 individuals, out of 459 who were invited to participate, responded to the survey (participation rate 43.6%). However, three were excluded because of a lack of baseline data, and four participants withdrew their consent to participate at a later phase of the study and were subsequently excluded from the dataset. The basic demographics of the participants are shown in Table 1. The mean age was 79.2 [SD: 6.51], and 78 (39.8%) were female. The mean number of medications was 8.04 (SD: 2.54). The number of participants reporting any falls, ER visits, and hospitalization in the past 6 months was 25 (12.8%), 24 (12.2%), and 51 (26.0%), respectively. Among all participants, 96 (49.2%) reported consulting their PCP about changing their medications in the past 6 months. Among these 96 participants, the majority did not have medication changed (57/96, 59.4%); those that had either increased or decreased number of medications were 10/96 (10.4%) and 27/96 (28.1%), respectively. Among the 57 participants who had their medication changed, 18/57 (31.6%) reported that their health status did not change, whereas 16/57 (28.1%) reported an improvement and 3/57 (5.3%) reported deterioration.

**TABLE 1:** Basic demographic of the participants.

	Number (%) or mean[SD]
Age	79.2 [6.51]
Gender	
Female	78 (39.8%)
Long‐term care insurance^a^	
Not receiving benefit	148 (76.3%)
Support level 1–2	29 (15.0%)
Care level 1–2	14 (7.2%)
Care level 3–5	3 (1.6%)
Residence	
Home	195 (99.5%)
Long‐term care facility	1 (0.5%)
Highest education	
Junior high school (9 years)	25 (13.0%)
High school (9–12 years)	85 (44.0%)
Higher education (12 or more years)	83 (43.0%)
Number of medications	8.04 [2.54]
Number of comorbidities	1.70 [1.23]
Comorbidities^b^	
Hypertension	111 (56.6%)
Diabetes	48 (24.5%)
Post‐MI, angina	37 (18.9%)
Cancer	35 (17.9%)
Dyslipidemia	31 (15.8%)
Chronic renal failure	21 (10.8%)
Chronic heart failure	19 (9.7%)
Dementia	11 (5.6%)
Asthma	7 (3.6%)
Liver disease/cirrhosis	5 (2.6%)
Depression	5 (2.6%)
COPD	3 (1.5%)
Hospitalization^c^	51 (26.0%)
ER visit^c^	24 (12.2%)
One or more falls^c^	25 (12.8%)
Subjective health compared to peers	
Excellent	8 (4.1%)
Very good	11 (5.6%)
Good	53 (27.0%)
Same as others	68 (34.7%)
Bad	56 (28.6%)

Abbreviations: chronic obstructive pulmonary disease; ER, emergency room; COPD, MI, myocardial infarction.

^a^ Care level is reported as defined by the Japanese Long‐term care insurance. Those not receiving benefit are independent, and care dependency increases until level 5 where beneficiaries are largely bedbound.

^b^ Comorbidities were self‐reported, where participants had to answer either yes or no to 12 conditions listed in the table. The number of comorbidities was obtained as the sum of comorbidities listed above that the participant answered “yes.”

^c^ Hospitalization, ER visit and falls were self‐reported for the past 6 months.

The numbers and percentages of answers, as well as the mean and SD for each question, are shown in Table 2. Floor effects and ceiling effects were not observed for any of the items.

**TABLE 2:** Number and percentages of respondents for each answer.

		*N* (%) of responses					Mean [SD]
		1	2	3	4	5	
1	I sometimes worry about becoming too dependent on my medicines.	34 (17.4%)	63 (32.1%)	44 (22.5%)	52 (26.5%)	3 (1.5%)	2.63 [1.10]
2	I feel that I am taking a large number of medicines.	18 (9.2%)	28 (14.3%)	47 (24%)	94 (48%)	9 (4.6%)	3.24 [1.06]
3	I am comfortable with the number of medicines that I am taking.	11 (5.6%)	86 (43.9%)	75 (38.3%)	24 (12.2%)	(0%)	2.57 [0.78]
4	I believe one or more of my medicines is giving me side effects, unwanted reactions, or other problems.	61 (31.1%)	75 (38.3%)	40 (20.4%)	19 (9.7%)	1 (0.5%)	2.10 [0.97]
5	I am taking one or more medicines that I would like to stop.	39 (19.9%)	55 (28.1%)	61 (31.1%)	38 (19.4%)	3 (1.5%)	2.55 [1.06]
6	I feel that I may be taking one or more medicines that I no longer need.	43 (21.9%)	78 (39.8%)	47 (24%)	27 (13.8%)	1 (0.5%)	2.31 [0.98]
7	All in all, I have complete trust in my PCP.	3 (1.5%)	7 (3.6%)	19 (9.7%)	101 (51.5%)	66 (33.7%)	4.12 [0.84]
8	My PCP is totally honest with me.	1 (0.5%)	5 (2.6%)	17 (8.7%)	110 (56.1%)	63 (32.1%)	4.17 [0.73]
9	How much effort does your PCP make to listen to the things that matter most to you when it comes to taking medicines?	5 (2.6%)	21 (10.7%)	39 (19.9%)	99 (50.5%)	32 (16.3%)	3.67 [0.96]
10	My PCP knows a lot about all of my medicines.	2 (1%)	9 (4.6%)	28 (14.3%)	108 (55.1%)	49 (25%)	3.98 [0.82]
11	My PCP knows about all of my medical problems.	(0%)	17 (8.7%)	44 (22.5%)	99 (50.5%)	36 (18.4%)	3.79 [0.84]

*Note*: For all questions except questions number 3 and 9, answer options were 1: strongly disagree, 2: disagree, 3: neutral, 4: agree, 5: strongly agree. For question 3, they were: 1: strongly agree, 2: agree, 3: neutral, 4: disagree, 5: strongly disagree. For question 9, they were: 1: no effort, 2: little effort, 3: some effort, 4: a lot of effort, 5: every effort.

Abbreviations: %, percentage; *N*, number; PCP, primary care physician.

### 3.3 Internal consistency, construct, and convergent validity

The Cronbach's alpha coefficients for subscales 1 and 2 were 0.763 and 0.767, respectively.

**FIGURE 2:**
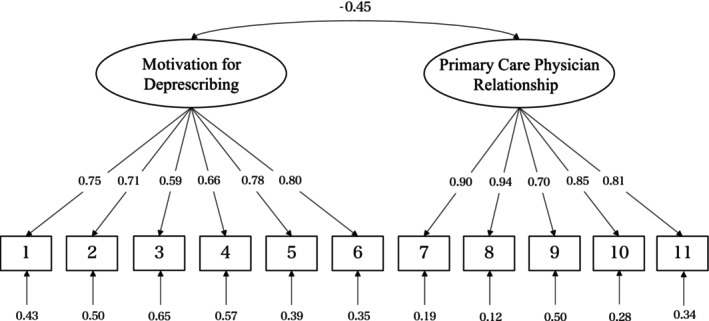
Path diagrams of the confirmatory factor analysis used to assess the structural validity. Squares represent the observed variable (questions 1–11), and ellipses represent the subscales. Single‐headed arrows from the ellipses to the squares represent factor loadings, the single‐headed arrow below each square represents residuals, and the double‐headed arrow represents factor correlations.

Figure 2 shows the path diagrams of the confirmatory factor analysis used to assess the structural validity of the Japanese SF‐PPoD. All factor loadings were above 0.40, indicating an acceptable correlation between the item and the factor. The confirmatory factor analysis showed a reasonable fit (CFI = 0.921, TLI = 0.899, SRMR = 0.059, and RMSEA = 0.112).

In order to test for convergent validity, we obtained the mean and SD of PPoD criteria‐based scores for subgroups divided by their experience of deprescribing in the past 6 months. The results are shown in Table 3. Participants who consulted their PCPs about medication change and who reduced medications tended to have higher readiness to deprescribe than those who did not consult their PCPs about medication change, although these differences were not statistically significant (*p* = 0.173 for subscale 1 and *p* = 0.57 for subscale 2). Even when comparing those who did not consult versus those who consulted regardless of the outcome, the PPoD‐SF scores did not have a statistically significant difference (*p* = 0.093 for subscale 1 and *p* = 0.414 for subscale 2).

**TABLE 3:** Mean and SD of scores for subgroups divided by their previous experience in deprescribing.

	Overall (*N* = 196)^a^		No consultation about medication change (*N* = 99)		Consulted but had no change (*N* = 67)^b^		Consulted and had reduced medications (*N* = 27)		*p* Value^c^
	Mean	SD	Mean	SD	Mean	SD	Mean	SD	
Subscale 1 Motivation for deprescribing	15.4	4.4	14.9	4.3	15.6	4.3	16.7	4.3	0.173
Subscale 2 Primary care physician relationship	19.7	3.4	19.5	3.6	19.9	3.5	20.2	2.4	0.57

^a^ All participants (*N* = 196) were included for the overall score mean. Three participants lacked data on their consultation status, thus excluded from the grouping.

^b^ Among participants who had consulted their PCP about changing their medication, those who either had an increase or did not have a change are included in this group.

^c^ *p* Value was obtained by ANOVA of the three subgroups (no consultation, consulted but had no change, and consulted and had reduced medications).

### 3.4 Intra‐rater reliability

Among 93 participants in the retest, the intraclass correlation coefficient (ICC) for subscales 1 and 2 were 0.60 and 0.62.

## 4 DISCUSSION

In this study, we developed a Japanese version of SF‐PPoD, which demonstrated adequate validity, internal consistency, and intra‐rater reliability using processes compliant with COSMIN guidelines. Overall, the Japanese SF‐PPoD is a psychometrically valid instrument for measuring patients' readiness for deprescribing.

Confirmatory factor analysis without modification showed a CFI of 0.921, slightly below the criteria of 0.95 set a priori, and RMSEA of 0.112, which was larger than the a priori criteria of 0.06.^36^ However, combined with the favorable TLI and SRMR, the evidence overall supports the construct validity of the scale. Although some results depart from thresholds recommended by COSMIN,^35^ it aligns with previous studies, including the validation of the original SF‐PPoD.^16^, ^36^

The newly developed Japanese PPoD‐SF holds promise for multiple stakeholders within the healthcare field. For clinicians, this tool can be used to better identify patients who are ready for deprescribing. The scale consists of 11 simple questions, making it adaptable to busy clinical settings. For researchers, the utility of this instrument extends to future research in the field to clarify patient factors associated with readiness to deprescribe or to investigate effective interventions to enhance patient readiness. Furthermore, because the original version is validated in the US context, the tool can be used for cross‐cultural comparison of readiness to deprescribe.

### 4.1 Strengths of this study

There are several strengths of this study. First, we developed the Japanese SF‐PPoD in accordance with the principles reported by the ISPOR task force for translation and cultural adaptation.^27^ Second, we used a robust method, strictly aligned with the COSMIN methodology, for systematic reviews of Patient‐Reported Outcome Measures.^35^ All analyses, including the test–retest, showed good internal consistency, intra‐rater variability, and construct validity. Content validity, especially face validity, was ascertained by the coauthors, who included researchers in this area, a PCP practicing in Japan, and the developer of the original PPoD. Thirdly, we suggest that this scale is well‐received and highly acceptable. We performed a cognitive debriefing and assessed it based on benchmarks for cultural adaptation, which showed good responsiveness and interpretability.

Furthermore, another strength of the Japanese SF‐PPoD is that we ensured cultural sensitivity. Because patients in Japan do not need referrals from their PCP to see other providers, it is not uncommon for patients to seek care from different providers at the same time. Thus, terms such as PCP may be difficult to understand. In this study, the review team, including the Japanese PCP, ensured that the terms were easily understood in the Japanese context. The survey was then administered only to participants who had a PCP, thus ensuring the validity of the tool among Japanese people who have a PCP. However, we asked for the respondents' self‐perceived PCP and could not evaluate whether there was more than one provider prescribing medicines for the respondents.

### 4.2 Limitations of the study

There are several limitations to this study. First, the results may not be generalizable to suburban or remote areas of Japan, for this study was conducted in healthcare facilities from urban areas. Although we included both a clinic and a general hospital in the study settings, patient demographics and values may differ in other Japanese settings. Second, non‐response bias may have affected the results. It is possible that participants who gave consent to participate in this study had positive perceptions toward their PCP. Thus, scores for questions about trust in PCP may have been different among responders and non‐responders.

Also, we could not test the criterion validity, for there is no gold‐standard test to measure readiness for deprescribing. Whether this tool is useful in routine clinical settings also needs further investigation. Finally, further study is needed to ascertain the usefulness of this tool in predicting success in deprescribing.

### 4.3 Conclusions

We developed and validated a Japanese version of the SF‐PPoD, with an 11‐item, two‐dimensional structure consistent with the original SF‐PPoD. This scale may facilitate shared decision‐making for medication optimization among older adults taking multiple medications while enhancing cross‐cultural comparison regarding patient perception and its relationship with deprescribing.

## FUNDING INFORMATION

The funding for this study was provided by the Ministry of Education, Culture, Sports, Science and Technology, Grant‐in‐Aid for Young Scientists, 2022–2023 (grant number 22K15678). The funding source had no role in the design, practice or analysis of this study.

## CONFLICT OF INTEREST STATEMENT

KI has received grant funding from the Japan Agency for Medical Research and Development (AMED) outside the submitted work (grunt number 22FK0108512H001). AML has received grant funding from VA Health Systems Research and her time is supported by resources from the VA Boston Healthcare System. She has received compensation from the US Deprescribing Research Network and AHRQ for deprescribing related work. MK, MT, and SMA: none to declare. KI is an Editorial Board member of the Journal of General and Family Medicine and a co‐author of this article. To minimize bias, he was excluded from all editorial decision‐making related to the acceptance of this article for publication.

## ETHICS STATEMENT

Ethics approval statement: The study protocol was approved by the Institutional Ethical Committee of St. Marianna University School of Medicine (No. 5593).

Patient consent statement: Written informed consent was obtained from all study participants.

Clinical trial registration: None.

## Supporting information


Tables S1–S2

## Data Availability

The datasets generated during the study will be available from the corresponding author upon reasonable request.
