# Supplementary material for: Development of a Japanese version of the patient perceptions of deprescribing – Short form
Source: J Gen Fam Med. 2024 Oct 6;26(1):45–53. doi: 10.1002/jgf2.733 (PMC11702458; doi:10.1002/jgf2.733)
Supplement: Supplementary file 1 — Tables S1–S2 [file JGF2-26-45-s001.docx]

**Supplemental Table 1: The original, translated and back-translated versions of the PPoD-SF questions.**

**■Subscale 1: Motivation for deprescribing**

1. I sometimes worry about becoming too dependent on my medicines

translation: 薬に依存しすぎるのではないかと心配になることがある

back-translation: I get worried that by taking the medicines I might become dependent on any of my medicines

2. I feel that I am taking a large number of medicines

translation:自分が飲んでいる薬の数が多いと感じることがある

back-translation: I feel that the number of medicines I take is too much

3. I am comfortable with the number of medicines that I am taking

translation:自分が服用している薬の数に納得している

back-translation: I am satisfied with the number of medicines I have to take every day

4. I believe one or more of my medicines is giving me side effects, unwanted reactions, or other problems

translation:自分が服用している薬のうち、一つ以上の薬が副作用や好ましくない反応、その他の問題を引き起こしていると思う

back-translation: I think that at least one of the medicines I take is causing adverse effects, undesirable reactions, or any other types of problems with my health.

5. I am taking one or more medicines that I would like to stop

translation:今飲んでいる薬の中で、やめたいと思うものが1つ以上ある

back-translation: I have one or more medicines among my prescribed medicines that I want to quit taking.

6. I feel that I may be taking one or more medicines that I no longer need

translation:自分には必要のない薬を飲んでいる可能性があるように感じる

back-translation: I feel that I might be taking medicines that I do not need.

**■Subscale 2: Primary care physician relationship**

7. All in all, I have complete trust in my PCP

translation:全体的に見て、私は自分のかかりつけ医を完全に信頼している

back-translation: Generally speaking, I fully trust my primary care physician

8. My PCP is totally honest with me

translation:私のかかりつけ医は私に対して本当に誠実だ

back-translation: My primary care physician is truly sincere with me

9. How much effort does your PCP make to listen to the things that matter most to you when it comes to taking medicines?

translation:あなたのかかりつけ医は、薬の服用に際してあなたにとって最も重要なことを聞く努力をどの程度してくれますか？

back-translation: How hard does your primary care physician try to understand what is most important for you in taking your medicines?

10. My PCP knows a lot about all of my medicines

translation:私のかかりつけ医は、私が飲んでいる全ての薬についてよく知っている

back-translation:  My primary care physician knows very well about every medicine I take.

11. My PCP knows about all of my medical problems

translation:私のかかりつけ医は、私の健康問題の全てを知っている

back-translation: My primary care physician knows well about all of my health issues.

**Supplemental table 2: The finalized version of Japanese PPoD-SF.**

この調査の目的は、あなたの薬に関する経験や、薬についてどのように考えているかを知ることです。このアンケートに答えるとき、あなたが普段かかっているかかりつけ医との経験について思い浮かべてください。この調査では、その医師をあなたの「かかりつけ医」と呼びます。

１）お薬について
あなたが日頃飲んでいる薬について思い浮かべてください。
以下の各項目について、最も当てはまる回答を１つ選択してください。

１. 薬に依存しすぎるのではないかと時々心配になることがある

どちらとも
いえない

そう思わない

全くそう

思わない

非常に
そう思う

そう思う

□　　　　　　　　　　　　□　　　　　　　　　　　　□　　　　　　　　　　　　□　　　　　　　　　　　　□

２. 自分が飲んでいる薬の数が多いと感じることがある
□　　　　　　　　　　　　□　　　　　　　　　　　　□　　　　　　　　　　　　□　　　　　　　　　　　　□

どちらとも
いえない

そう思わない

全くそう

思わない

非常に
そう思う

そう思う

３. 自分が服用している薬の数に納得している
□　　　　　　　　　　　　□　　　　　　　　　　　　□　　　　　　　　　　　　□　　　　　　　　　　　　□

どちらとも
いえない

そう思う

非常に
そう思う

全くそう
思わない

そう思わない

４. 自分が服用している薬のうち、１つ以上の薬が副作用や好ましくない反応、その他の問題を引き起こしていると思う
　　　　□　　　　　　　　　　　　□　　　　　　　　　　　　□　　　　　　　　　　　　□　　　　　　　　　　　　□

そう思う

非常に
そう思う

全くそう

思わない

そう思わない

どちらとも
いえない

５. 今飲んでいる薬の中で、やめたいと思うものが１つ以上ある
□　　　　　　　　　　　　□　　　　　　　　　　　　□　　　　　　　　　　　　□　　　　　　　　　　　　□

どちらとも
いえない

そう思わない

全くそう

思わない

非常に
そう思う

そう思う

６. 自分には必要のない薬を飲んでいる可能性があるように感じる
□　　　　　　　　　　　　□　　　　　　　　　　　　□　　　　　　　　　　　　□　　　　　　　　　　　　□

どちらとも
いえない

そう思わない

全くそう

思わない

非常に
そう思う

そう思う

２）医療者について

あなたのかかりつけ医についてお聞きしたいと思います。

過去１年間にこのかかりつけ医にかかった全ての受診を思い浮かべて、以下の各項目について、最も当てはまる回答を１つ選択してください。

７. 全体的にみて、私は自分のかかりつけ医を完全に信頼している
□　　　　　　　　　　　　□　　　　　　　　　　　　□　　　　　　　　　　　　□　　　　　　　　　　　　□

どちらとも
いえない

そう思わない

全くそう

思わない

非常に
そう思う

そう思う

８. 私のかかりつけ医は私に対して本当に誠実だ
□　　　　　　　　　　　　□　　　　　　　　　　　　□　　　　　　　　　　　　□　　　　　　　　　　　　□

どちらとも
いえない

そう思わない

全くそう

思わない

非常に
そう思う

そう思う

９. あなたのかかりつけ医は、薬の服用に際してあなたにとって最も重要なことを聞く努力をどの程度してくれますか？
　　　　□　　　　　　　　　　　　□　　　　　　　　　　　　□　　　　　　　　　　　　□　　　　　　　　　　　　□

よく
してくれる

あらゆる努力をしてくれる

全くしない

少しだけ
してくれる

いくらか
してくれる

１０. 私のかかりつけ医は、私が飲んでいる全ての薬についてよく知っている
□　　　　　　　　　　　　□　　　　　　　　　　　　□　　　　　　　　　　　　□　　　　　　　　　　　　□

どちらとも
いえない

そう思わない

全くそう

思わない

非常に
そう思う

そう思う

１１. 私のかかりつけ医は、私の健康問題の全てを知っている
□　　　　　　　　　　　　□　　　　　　　　　　　　□　　　　　　　　　　　　□　　　　　　　　　　　　□

どちらとも
いえない

そう思わない

全くそう

思わない

非常に
そう思う

そう思う
